# Computing with biological switches and clocks

**DOI:** 10.1007/s11047-018-9686-x

**Published:** 2018-06-01

**Authors:** Neil Dalchau, Gregory Szép, Rosa Hernansaiz-Ballesteros, Chris P. Barnes, Luca Cardelli, Andrew Phillips, Attila Csikász-Nagy

**Affiliations:** 1 Microsoft Research, Cambridge, UK; 2 King’s College London, London, UK; 3 University College London, London, UK; 4 University of Oxford, Oxford, UK; 5 Pázmány Péter Catholic University, Budapest, Hungary

**Keywords:** DNA computing, Systems biology, Synthetic biology, Distributed computing, Oscillation, Bistability, Feedback loop, Network

## Abstract

The complex dynamics of biological systems is primarily driven by molecular interactions that underpin the regulatory networks of cells. These networks typically contain positive and negative feedback loops, which are responsible for switch-like and oscillatory dynamics, respectively. Many computing systems rely on switches and clocks as computational modules. While the combination of such modules in biological systems leads to a variety of dynamical behaviours, it is also driving development of new computing algorithms. Here we present a historical perspective on computation by biological systems, with a focus on switches and clocks, and discuss parallels between biology and computing. We also outline our vision for the future of biological computing.

## History of understanding biological computation

In the last 50 years, biology has inspired computing in several ways (Navlakha and Bar-Joseph 2011; Cardelli et al. 2017). During this time, computational thinking has also improved our understanding of biological systems (Bray 1995; Goldbeter 2002; Nurse 2008). Using principles from chemistry, physics and mathematics, we have understood that the highly complex behaviour of biological systems is caused by a multitude of coupled feedback and feed-forward loops in the underlying molecular regulatory networks (Alon 2007; Tyson and Novák 2010). In particular, we have learned that positive and negative feedback loops are responsible for driving biological switches and clocks, respectively (Tyson et al. 2008). We have understood much about the behaviour of these basic units of biological computation (Ferrell 2002; Novák and Tyson 2008; Tyson et al. 2003) and simple switches and clocks have been synthesized in single cells more than 15 years ago (Becskei and Serrano 2000; Gardner et al. 2000; Elowitz and Leibler 2000). Nevertheless, we still lack a comprehensive understanding of how these computational modules have emerged, and which features and molecular interactions are responsible for their efficient and robust behaviour (Cardelli et al. 2017). Ideas from computing might help us to take this last step, which might enable *biological* switches and clocks to be influential in the development of future computing technologies. The similarity between the biological switch controlling mitotic entry and the approximate majority algorithm of distributed computing (Angluin et al. 2008; Cardelli and Csikász-Nagy 2012) suggests that computing and molecular biology could further influence each other in the future. With the emergence of the fields of systems and synthetic biology, there has been increased interaction between computer science and biology, but there are a few steps needed before we can realise a biology-inspired soft-matter computational revolution. In this paper we review some of the key advances we have seen as a result of the interplay between computing and biology and speculate on the directions that a possible joint field could take in the near future.

### Computation

The basic components of computational devices, including modern electronic circuits and earlier mechanical equivalents, consist mainly of Boolean and arithmetic functional units (Boolean control logic, integer and floating point units, analog to digital converters, etc.), of registers to hold intermediate results of iterative algorithms, and of coordination components that orchestrate the flow of information across registers and functional units. Coordination is most often achieved by clocks: at each tick data is frozen into registers, and between ticks data flows between registers through the functional units. This is the so called von Neumann architecture that, despite dramatic technology improvements and architectural refinements, has remained largely unchanged since the first electronic computers.

Functional units compute Boolean and mathematical functions by combinational logic (that is, without requiring memory or timing coordination). We can easily find analogues of these in biology, like the function computed by the regulatory region of a single gene (Arnone and Davidson 1997). Synthetic biology has demonstrated how many such functions, typically Boolean gates, can be engineered in vivo by a variety of genetic and protein-based mechanisms (Siuti et al. 2013). More theoretically, it has been shown how chemical reaction networks can compute complex functions (Buisman et al. 2009). Although much of this work has mimicked digital components, there is a sentiment that functional units in biology work mostly in the analog domain, and that synthetic biology could benefit from this approach (Sauro and Kim 2013).

In this review we focus mainly on the other two classes of components: memory and coordination. A switch is a memory unit capable of storing a single bit: at the core there is a bistable dynamical systems coupled with a mechanism to force the system from one stable state to the other. Switching behaviour is pervasive in biology: it is achieved by a range of mechanisms, from individual molecular components like phosphorylation sites and riboswitches, to whole complex biochemical networks that switch from one configuration to another, such as in the cell cycle switch. Synthetic genetic switches have also been demonstrated (Gardner et al. 2000).

The intricate feedback loops of biochemical networks tend to produce oscillations in abundance, both stable and transient, many of which are poorly understood. The most prominent oscillators in biology, found also in the most primitive organisms, are those involved in the cell cycle and in circadian clocks, whose cyclic activities coordinate much of cellular function. Oscillations can also be observed in systems consisting of just 1 to 3 proteins, as in the case of the KaiC circadian oscillator (Nakajima et al. 2005), although those proteins have a very sophisticated structure. Theoretically, many chemical oscillators consisting of 2 to 3 simple species have been studied (Bayramov 2005).

Although similar basic components (switches, oscillators, and functional units) are found both in biology and in computer engineering, it does not necessarily mean that these systems compute “in the same way”. In particular, coordination is achieved in fundamentally different ways in biological systems than in the von Neumann architecture. In biology, oscillators coordinate events only at the coarsest level of granularity, while fine-grained coordination is achieved by direct interaction between molecular components. In the central processing unit of computers, oscillators instead coordinate events at the finest grain, and do so at great cost. As a result, low-power devices tend to employ clock-free coordination strategies to save power. At the level of computer networks, though, coordination is achieved by message passing, because individual clocks can get out of step and network latency may vary. Many non-von Neumann models of computation have been studied in the area of distributed computing: these models resemble, and sometimes even technically coincide, with biochemical models (Angluin et al. 2006; Chen et al. 2014).

The general architecture of computation in biochemical systems is still a matter of investigation, and so is the functioning of many subsystems that appear to process information. For the moment we can focus on how nature achieves the functionality of the basic components, switches, oscillators and functional units, while using material and constraints that are very different from those that come from engineering.

### How do natural systems compute?

The complex dynamics of natural systems drew research interest a long time ago. The theory of dynamical systems and chaos was born at the turn of the twentieth century, with a focus on understanding the weather and the many-body problem (Strogatz 2000). Pioneers of mathematical modelling of biological systems came from the field of chemical physics and used their experience learned from non-equilibrium chemical systems to investigate biological switches and clocks (Goldbeter 2017). Ideas on the chemical basis of biological behaviour were also used by the computer scientist Alan Turing to explain developmental pattern formation (Turing 1952). Yet still computing had far less influence on our thinking about biological systems than chemistry, physics or mathematics. Indeed, biological behaviour is controlled by (bio)chemical reactions and the underlying reaction kinetics can be understood by looking at the microscopic physical behaviour of molecules, but to turn these into a comprehensive form, mathematical expertise is required. Since the 1990s, advances in computing have enabled us to solve highly complex equations describing the physical interactions of the chemical reactions driving biological behaviour, but it was the appearance of systems biology (Kitano 2002a) that led to the understanding that we need more computing to truly understand biological systems (Kitano 2002b). Data-rich biological experiments at the molecular level have identified the ubiquity of switches and clocks (Goldbeter 2002) as core components of complex biological regulatory networks.

#### Feedback loops

Already by the 1960’s it was known that feedback loops are the key determinants of the dynamics of biological systems (Griffith 1968a, b). Positive feedback loops are key to the appearance of switching behaviour, while negative feedback loops are needed for oscillations (Ferrell 2002; Goldbeter 2002). The complex dynamics of biological systems is determined by the combination of multiple of such feedback loops (Tyson et al. 2003). Here we present the main features of feedback loops that enables them to drive key biological processes.

**Fig. 1:**
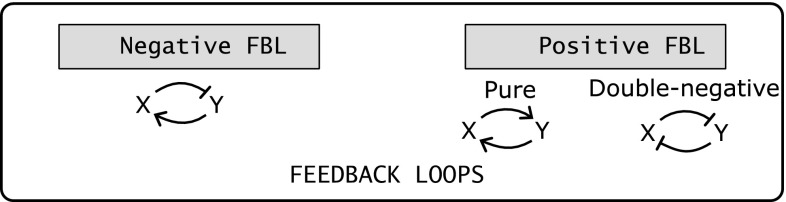
Examples of Feedback loops. Left, a negative feedback loop composed of two molecules. Right, a pure positive feedback loop is formed by only positive interactions, while a double-negative feedback loop contains an even number of negative interactions

Feedback loops (FBLs) arise when at least two molecular species regulate each other’s activity (Fig. 1). There are two types of FBLs, negative or positive. Negative FBLs (NFBLs) appear when the production or activation of a species is either directly or indirectly repressed when this same species is active (autoregulation) (Thomas and D’Ari 1990; Thomas et al. 1995). Negative feedback loops contain an odd number of inhibitions. In Fig. 1, a system of only two components is shown, where one of the molecular species (X) exhibits inhibitory activity over the other (Y), while this other molecule Y is activating the first molecule X.

Positive FBLs (PFBLs) auto-enhance the production of the species involved in the loop. There are two subtypes of PFBL, pure positive or double-negative. Pure PFBLs contain only interactions of activation, while double-negative PFBLs, or antagonistic interactions, contain an even number of inhibitions (plus any number of activations). (Fig. 1).

Feedback loops (FBLs) constitute a basic relationship between molecular species to construct complex behaviours and consequently are abundant in protein regulatory networks. FBLs can produce various dynamical behaviours, such as efficient switching and oscillations (Thomas et al. 1995; Thomas 1981; Tyson et al. 2003; Tyson and Novák 2010; Hernansaiz-Ballesteros et al. 2016; Cardelli et al. 2017). Switch-like dynamics requires PFBLs, producing two (or more) stable states of the system (usually on/off states), when a given species is either fully active or inactive. This feature of PFBLs is known to be key for developmental and decision-making processes (Ferrell 2002). In contrast, oscillations require the presence of NFBLs. While direct negative feedback can stabilize a system, the introduction of a delay arising from regulation via an intermediate, or simply through a slow accumulation, can very easily lead to oscillations. If a system contains at least three different molecular species and a strong non-linearity, a damped or sustained oscillator may arise (Griffith 1968b). Systems with only two molecular species and without explicit time delays can also oscillate, but they require the presence of a PFBL, creating a switch that drives the oscillation. In contrast, the combination of positive feedbacks with the depletion of one of the species creates systems that can oscillate without an explicit negative feedback loop. These so-called *relaxation oscillators* produce characteristic fast switching in one direction, with slow switching in the other direction, producing triangular-like waveforms (Sel'Kov 1968). Finally, several natural oscillations are known to integrate positive and negative feedback loops, which is thought to enhance the oscillator network robustness to intrinsic or extrinsic fluctuations (Thomas 1981; Thomas et al. 1995; Novák and Tyson 2008; Ferrell et al. 2011).

#### Systems biology of switches and clocks

The importance of switches and clocks as basic modules of biological networks was highlighted at the birth of systems biology (Hartwell et al. 1999). Two contrasting approaches of systems biology modelling are (1) a top-down approach, where large-scale datasets are used to infer an underlying molecular regulatory network and (2) a bottom-up approach, where an abstract model of a regulatory system is derived from existing experimental data, and the model is subsequently tested against additional experimental data (Bruggeman and Westerhoff 2007). The bottom-up approach often involves models that combine feedback loops to explain complex dynamical behaviour, which often include a combination of switches and clocks (Tyson et al. 2003). Indeed, some of the earliest examples of cycles of model refinement and testing (Chen et al. 2000, 2004; Cross et al. 2002) came from the analysis of the cell cycle regulatory network, which combines two switches to control the major cell cycle transitions and an oscillator that is responsible for the periodicity of the process (Novák and Tyson 2008). Oscillators and switches were also shown to be important in the context of spatio-temporal control of cell signalling (Kholodenko 2006). Furthermore, the effect of the coupling between positive and negative feedback loops was also shown to be important for the robust periodicity of oscillators (Tsai et al. 2008). These and several other landmark papers have led to legitimate claims of *understanding* the functioning of these network motifs (Shoval and Alon 2010) and initial thinking about what could be the *algorithms* underlying cellular computation (Lim et al. 2013). In recent years, major steps have been taken to understand biological algorithms by synthesizing biological regulatory networks de novo, which aim to compute specific functions.

### Chemical reaction network design and synthetic biology

The advent of ever more precise genetic engineering requires an understanding of information processing in reaction-diffusion networks and harnessing the emergence of self-organising properties of such systems. Systems with switch-like and oscillatory behaviours have been a focus of synthetic biology for almost two decades. In a now classic Nature edition from 2000, the genetic toggle switch and the repressilator systems were described, which opened up a new field of biological engineering (Gardner et al. 2000; Elowitz and Leibler 2000). These systems not only serve as models for the engineering of complex emergent behaviours, but also allow us to test our hypotheses on how biological systems use feedback mechanisms within complex networks to function and perform computations. In the past few years, genetic switches and oscillators have also been used in a number of applications.

#### Synthetic switching systems

The classic genetic toggle switch used two mutually repressing transcription factors, which gives rise to bistablity and hysteresis (Gardner et al. 2000; Litcofsky et al. 2012). Subsequently, genetic switches were also constructed using positive autoregulatory feedback loops (Isaacs et al. 2003; Atkinson et al. 2003). More recently, circuits combining mutual repression with positive autoregulatory feedback have been built, including the addition of a single positive feedback loop (Lou et al. 2010) and double positive autoregulatory loops, resulting in a quadrastable switch (Wu et al. 2017). The genetic toggle switch has also been coupled with quorum sensing systems to create a population-based switch, which switched states dependent on the local cell density (Kobayashi et al. 2004). In bacterial cells, the cellular context is of increasing interest and this can affect genetic switch performance in a number of ways including changes in stability at low molecule numbers (Ma et al. 2012), plus dependence on host growth rate (Tan et al. 2009), sequence orientation (Yeung et al. 2017) and copy number (Lee et al. 2016). This suggests that natural systems have likely evolved mechanisms that are robust to some of these factors. However, gene regulatory networks are only one way to create switch-like behaviours. Alternatives include the use of recombinases, which allow the DNA itself to flip orientation (Friedland et al. 2009; Bonnet et al. 2012; Courbet et al. 2015; Fernandez-Rodriguez et al. 2015), and the use of transcriptional (RNA) systems (Kim et al. 2006). Accompanying theoretical and computational work has been equally diverse, with insights into possible network topologies (Angeli et al. 2004; Otero-Muras et al. 2012), stochasticity (Tian and Burrage 2006; Munsky and Khammash 2010; Jaruszewicz and Lipniacki 2013; Leon et al. 2016), robustness (Kim and Wang 2007; Barnes et al. 2011), time dependent transient behaviour (Verd et al. 2014), and emergent properties of populations of switches linked by quorum sensing (Kuznetsov et al. 2004; Wang et al. 2007; Nikolaev and Sontag 2016). Following the pioneering work in bacteria, there has now been an explosion of engineered switches for mammalian systems (see Kis et al. 2015 for a comprehensive review), which use components from diverse backgrounds (prokaryotic, eukaryotic and synthetic), and target a variety of applications.

#### Engineered biological oscillators

Synthetic genetic oscillators have undergone a number of significant developments. The original repressilator was constructed from three transcriptional repressor proteins arranged in a negative feedback cycle (Elowitz and Leibler 2000). Another topology that combined positive and negative feedback was first studied theoretically (Barkai and Leibler 2000) and then constructed in E. coli (Atkinson et al. 2003). An extension of this negative feedback oscillator, combining a further negative autoregulatory feedback loop, showed increased tunability and robustness (Hasty et al. 2002; Stricker et al. 2008). In a series of landmark papers, this network topology was coupled with quorum sensing to create populations of synchronised oscillators at different scales (Danino et al. 2010; Mondragón-Palomino et al. 2011; Prindle et al. 2012). This population-based circuit was eventually used for the treatment of tumours in mice, the oscillatory dynamics causing bacterial cells to lyse and release a chemotherapeutic agent directly into metastatic sites (Din et al. 2016). More recently, in an interesting development, the original negative feedback topology of the repressilator was revisited and re-engineered using detailed stochastic modelling to vastly improve its robustness, so much so that the oscillations remained synchronised without any need for quorum system interactions (Potvin-Trottier et al. 2016). Oscillators have also been implemented at the RNA level (Kim and Winfree 2011), metabolic network level (Fung et al. 2005), and in mammalian cells (Tigges et al. 2009, 2010). The theoretical properties of genetic oscillators have been studied extensively, including design principles (Guantes and Poyatos 2006; Novák and Tyson 2008), robustness (Wagner 2005; Ghaemi et al. 2009; Tsai et al. 2008; Woods et al. 2016; Otero-Muras and Banga 2016) and stochasticity (Vilar et al. 2002; Turcotte et al. 2008).

The engineering of biological systems in all organisms faces similar implementation challenges. Perhaps the main challenge is context dependence, which can occur at multiple levels (sequence, parts, evolutionary and environmental) (Cardinale and Arkin 2012; Arkin 2013). These include predictability of transcription and translation (Mutalik et al. 2013a, b); development of orthogonal part libraries (Wang et al. 2011; Nielsen et al. 2013; Chen et al. 2013b; Stanton et al. 2014); resource demand (burden, see later discussion); and impedance matching or retroactivity (balancing input sensitivity and output strengths) (Vecchio et al. 2008; Jayanthi et al. 2013). Eukaryotic systems offer additional challenges over prokaryotes due to their multi-cellularity, more complex genomes and higher levels of regulation (Ceroni and Ellis 2018). These challenges are increasingly being met with an interdisciplinary approach incorporating mathematical modelling, biochemistry, ‘omics’ approaches and ultimately a deeper understanding of the biology.

#### Synthetic biology and computation

Within the field of synthetic biology, a large body of work on computation has focussed on genetic Boolean gates (Moon et al. 2012). In this arena the state-of-the-art in transcription circuitry is the CELLO algorithm, which uses a characterised library of repressor proteins to design functional genetic implementations for any three-input Boolean circuit (Nielsen et al. 2016). Recombinases (Siuti et al. 2013) and the CRISPR/Cas system (Nielsen and Voigt 2014) can also be used to construct Boolean gates, and genetic Boolean circuits have also been combined with the toggle switch to create sequential logic operations (Lou et al. 2010), including a Pavlovian-like conditioning genetic circuit (Zhang et al. 2014). Most recently, work has shown that ribocomputing devices based on RNA operations can be used to create complex logic functions in living cells (Green et al. 2017). Notable examples of the translation of these approaches include cancer cell type discrimination (Xie et al. 2011) and immunotherapy (Nissim et al. 2017), both of which use Boolean logic computations on intracellular mRNA signals within mammalian cells.

The synthetic switches and oscillators described above have been used in a small number of non-Boolean computing applications inside living cells. For example, genetic switches have been used in signal processing applications including detecting small molecule signals in the mammalian gut (Kotula et al. 2014; Riglar et al. 2017) and glucose sensing (Chen and Jiang 2017). In another landmark study, coordination of genetic oscillators was achieved through coupling of post-translational processing of proteins (Prindle et al. 2014). External input signals in the form of chemical inducers and flow rate were encoded into frequency modulated oscillations. By exploiting the inherent queuing structure of protein degradation, both oscillators become coupled and the corresponding input signals combined into a single multispectral timeseries encoding both signals (Prindle et al. 2014). The theoretical study of multifunctionality in fixed network topologies has become of great interest recently (Jiménez et al. 2017) and work has shown that a genetic circuit comprising of both a toggle switch and a repressilator, known as the AC–DC circuit, has emergent properties such as coherent oscillations, excitability and spatial signal processing (Perez-Carrasco et al. 2018). These examples show that biological systems can be engineered to exploit feedback structures for analog and digital signal processing and that complex computations are possible at different scales. A computer science viewpoint of how biological systems process information and perform computation could help synthetic biology construct more complex systems, further elucidating how natural biological systems function.

Perhaps the most developed area of non-Boolean computing within synthetic biology is molecular programming, which uses nucleic acids (DNA, RNA) as the computational substrate. The use of DNA for computation was first introduced by Adelman to solve an instance of the Hamiltonian path problem (Adleman 1994). It worked by mapping DNA oligomers to edges between nodes in a small network and exploiting the huge parallelism of $\sim 10^{19}$ molecules to compute all possible paths using repeated use of polymerase chain reaction (PCR). Finally, oligomers of the correct length and containing the correct start and end sequences were extracted, in principle providing solutions to this NP-complete problem. Furthermore, the number of oligomers required is linear in the size of the network. Since then, molecular programming has progressed significantly and two modern approaches will be discussed in detail in Sect. 3.

## Dynamical correspondence between biological and computing networks

**Fig. 2:**
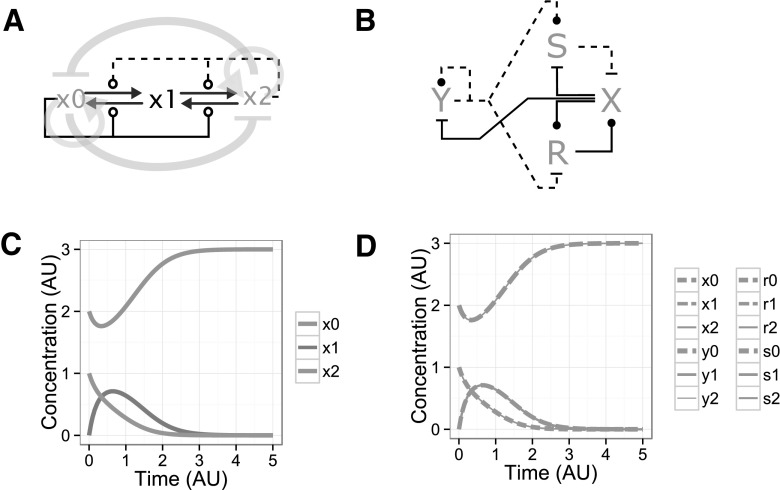
Structural morphism and emulation of AM by GW. **a**, **b** Wiring diagrams of Approximate Majority (AM) network and a system containing four species embedded in multiple feedback loops (GW). **a** The AM network shows the reactions involving the three forms of a single molecular species *X* (empty, ball ended arrows mean catalysis of a given reaction). **b** The GW network shows the interactions between four species. Arrows indicate the interactions between them (filled, ball-end means activation and dash-end means inhibition). **c, d** Simulation traces of AM and GW show that they follow exactly the same dynamics and that the individual species in GW (X, R, Y, S) can be mapped to the different forms of AM. Active forms of X and R ($x_0$ and $r_0$) and inactive forms of Y and S ($y_2$ and $s_2$) collapse into $x_0$ from AM. Inactive forms of X and R ($x_2$ and $r_2$) and active forms of Y and S ($y_0$ and $s_0$) collapse into $x_2$ from AM. All intermediary forms ($_1$) collapse with the intermediary form of AM. The similarity between the networks of panel A and B represent a *structural morphism* and the similarity of their dynamics mean *network emulation* exists between these two systems

The primary mathematical feature of a switch is bistability, which necessitates the existence of positive feedback loops. The simplest (bimolecular) chemical reaction network that realizes a robust bistable switch is the *Approximate Majority* (AM) network, a system based on competing autocatalytic feedback loops (Fig. 2a).

The name Approximate Majority comes from its origin in distributed computing, where the algorithm is used by a population of agents to reach consensus over one of two beliefs (states) that each agent can independently adopt (Angluin et al. 2008). At steady state, the whole population reaches the belief state that was initially in majority, but only approximately, since the algorithm is inherently stochastic. Nevertheless, it has been shown that this algorithm asymptotically optimally convergences to one of two stable steady states in $\mathcal {O}(\log n)$ time with high probability (Angluin et al. 2008), where *n* is the population size (number of agents). Moreover, the steady states are robust to large perturbations, and they are reached quickly even when starting from ambiguous configurations (Angluin et al. 2008). A third (undecided) state is critical for the functionality of the algorithm. Mapping this protocol to a biochemical reaction network produces a system described by 3 species (one per belief state) and 4 reactions (Fig. 2a). Initialised with *n* total molecules, this reaction network enjoys the same $\mathcal {O}(\log n)$ convergence. While this basic network exhibits only the bistability aspect of a switch, external controls can be added to flip the system from one state to the other.

In the biological literature, the exact interaction pattern of AM can be found in epigenetic switches, where DNA histones can be in one of three states: (M)ethylated, U(nmodified), or (A)cetylated (Dodd et al. 2007). A contiguous stretch of DNA consists of a population of histones that should be uniformly methylated or acetylated. This is achieved by the M and A states activating two proteins each that catalyse transitions between M–U–A states through the whole population. The known properties of AM imply robust uniform settling of the whole histone population into either M or A states, which is also the interpretation suggested in Ref. Dodd et al. (2007).

Many other biological switching systems employ systems that can be related to the AM algorithm. Usually these appear in a less direct way, with multiple species involved in the feedback loops. Even though these more complicated systems may look quite different, it is possible to apply a model reduction technique that maps them down to the basic AM network (Cardelli 2014). Similar reductions can be shown for various models of the cell cycle switch (Cardelli and Csikász-Nagy 2012), and can be summarized as follows.

A trajectory is a time-course evolution of the concentration of a single species. A complex network *emulates* a simpler network if it can reproduce all the possible trajectories of the simpler network, species by species, in the following sense: for any initial conditions of the simple network, there are initial conditions of the complex network such that the set of trajectories of the complex network is (with replications) exactly the same as the set of trajectories of the simple network (Fig. 2c,d). In short, emulation holds if the complex network can *always* mimic the simple network. Moreover, this emulation condition on trajectories, which is predicated on all possible initial states, can be shown to hold just by examining the structure of the networks (including rates and stoichiometry, but without considering initial conditions). In such a fashion it can be shown that various idealized cell cycle switches emulate AM (Cardelli and Csikász-Nagy 2012; Cardelli 2014). For instance, the Greatwall network (GW) (Cardelli and Csikász-Nagy 2012) summarizes the interactions of the cell cycle regulators Cdk, Wee1, Cdc25 and PP2a, corresponding to species X, S, R and Y respectively (Fig. 2b). This network can *emulate* the behaviour of the AM network (Fig. 2c,d).

### From switch-like behaviours to oscillatory dynamics

**Fig. 3:**
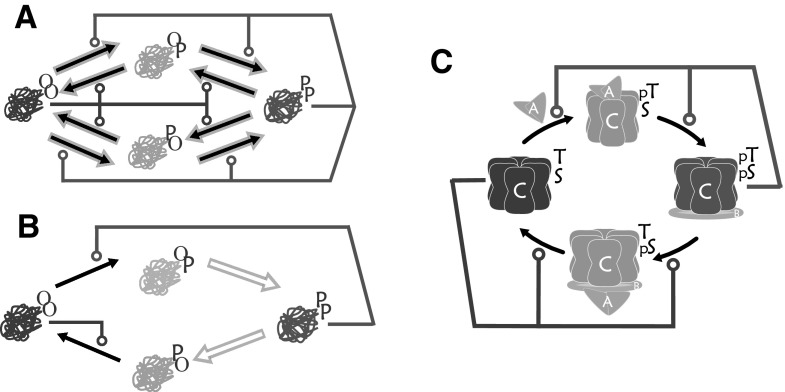
A simple switch turned into an oscillator. **a** The Two Intermediates (TI) system, that is behaving like a switch and emulating the behaviour of AM. **b** The Spontaneous Oscillator (SO) system is derived from TI and functions as robust oscillator. Solid arrows represent catalytic transitions with ball end arrows showing the activator of transitions, grey empty arrows represent first order conversions. **c** Representation of the KaiABC system of the cyanobacteria circadian clock (Loza-Correa et al. 2010), where KaiC hexamers are helped to convert themselves between forms that are phosphorylated an unphosphorylated at two critical sites (labelled T and S). KaiA (blue triangle) facilitates the phosphorylation reactions, while KaiB (yellow rod) helps the dephosphorylation reactions. (Color figure online)

Switch-like dynamics are widely exploited to control biological systems requiring memory and decision making (Tyson and Novák 2010). The AM system can efficiently function as a bistable switch and its dynamics can be emulated by a large class of complex biological networks (Cardelli 2014). AM works with a single undecided state, but in a real molecular system, the two modifications that lead to the two active forms of AM might not affect the same site. This led us to consider a Two Intermediates (TI) system with two intermediates (OP and PO) between the autocatalytic forms (OO and PP) (Fig. 3a). OO and PP can convert these species between the various forms producing a specific pattern of modification: $OO \longleftrightarrow OP \longleftrightarrow PP \longleftrightarrow PO \longleftrightarrow OO$. From a dynamical systems point of view, the TI system produces switch-like behaviour and emulates AM. In a biological context, this system has been proposed to function as a primitive sensor of the source of energy (Hernansaiz-Ballesteros et al. 2018). When energy level is above a critical threshold TI will be in the fully modified state PP, while as the energy source decreases the system will switch to the unmodified state OO.

The TI system maintains its toggle-switch behaviour even if some of the reaction paths are blocked (Hernansaiz-Ballesteros et al. 2018). The switching behaviour is lost only when at least two reactions are removed, which then results in oscillatory behaviour. The Spontaneous Oscillator (SO) network (Fig. 3b) is the simplest oscillatory system that can be reached from TI by removal of reaction paths (Hernansaiz-Ballesteros et al. 2018). The oscillations of SO autonomously follow the path $OO \rightarrow OP \rightarrow PP \rightarrow PO \rightarrow OO$ (Fig. 3b). Curiously, the SO network is remarkably similar to a well-known biological oscillator, the network driving the circadian clock of cyanobacteria (Fig. 3c). Here, the autocatalytic molecule KaiC, with the help of KaiA and KaiB, drives 24 h oscillations of phosphorylation cycles (Nakajima et al. 2005). It has been found that amongst the three components, KaiC is the most conserved, and sometimes appears in organisms without its two partners (Loza-Correa et al. 2010). Thus it was proposed that KaiC-like molecules in primitive organisms could have adopted a topology similar to either the TI or the SO systems, thus they could be working there either as switches or oscillators respectively (Hernansaiz-Ballesteros et al. 2018).

There is a critical interest in finding minimal networks that can serve crucial biological functions. AM was already shown to serve as a minimal switch (Cardelli and Csikász-Nagy 2012), and we can now see that SO could serve as a minimal clock (Hernansaiz-Ballesteros et al. 2018). As the two can be converted to one another through reaction duplications and reaction removals, there is some suggestion of an evolutionary link between these architectures. This gives us a hope that these and other related systems could be implemented in synthetic networks that can be used for complex biological or computing tasks.

## Molecular programming

Molecular Programming involves *“the specification of structures, circuits, and behaviours both within living and non-living systems–systems in which computing and decision-making are carried out by chemical processes themselves”* (molecular-programming.org). Nucleic acids are currently the molecules of choice for molecular programming, due to their high degree of programmability via Watson-Crick complementarity and their ability to directly interface with biological components, with potential applications in sensing, diagnosis and treatment of disease. A number of approaches have been proposed for implementing computation in nucleic acids. Here we will summarise two of the main ones—DNA Strand Displacement and PEN DNA Toolbox—and describe how they have been used to implement switches and clocks.

### DNA strand displacement

**Fig. 4:**
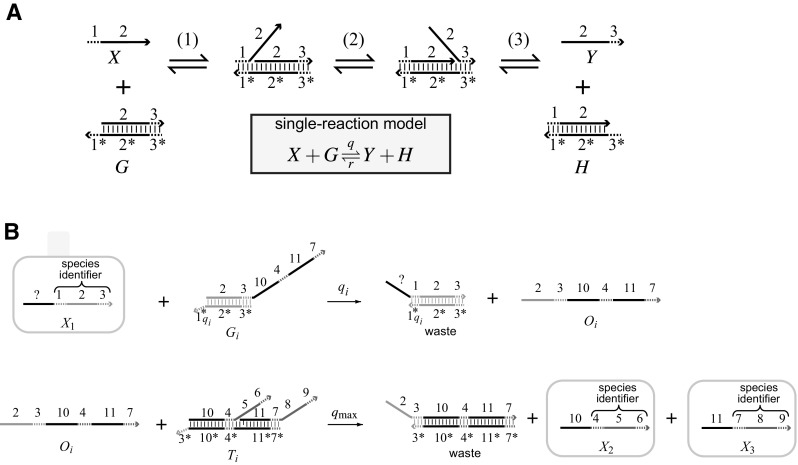
A 4-domain scheme for implementing chemical reaction networks in DNA, reproduced from Soloveichik et al. (2010). **a** An elementary DNA strand displacement interaction, modelled by the chemical reaction $X + G \rightarrow Y + H$. Chemical species *X* denotes a single DNA strand consisting of domains 1 and 2, where each domain corresponds to a DNA sequence. The strand X is written <1 2>, where the 3’ end of the strand is assumed to be on the right, represented graphically by an arrowhead. The species *G* denotes a complex consisting of strand <2 3> hybridized to the strand <3* 2* 1*>, where the star (*) denotes Watson-Crick complementarity. The reaction takes place in three steps: (1) The domain 1 binds to its complement 1*. The reaction is reversible, since the domain 1 is assumed to be short enough to spontaneously unbind. These short domains are referred to as *toeholds*; (2) The domain 2 of strand <1 2> displaces the domain 2 of strand <2 3> by a random walk process, referred to as *branch migration*; (3) The toehold domain 3 spontaneously unbinds from its complement 3*. **b** A 4-domain encoding of the formal unimolecular reaction $X_1 \rightarrow X_2 + X_3$, with reaction index *i*. The species of this formal reaction are represented as DNA strands and highlighted by boxes, with $X_1$ represented as < ? 1 2 3>, $X_2$ as <10 4 5 6> and $X_3$ as <11 7 8 9>. Black domains are assumed to be unique to each reaction *i*, while green, red and blue domains are associated to species $X_1$, $X_2$ and $X_3$, respectively. The formal reaction $X_1 \rightarrow X_2 + X_3$ is implemented by two DNA complexes, which are assumed to be present in excess and are consumed over time. The first complex $G_1$ binds the species $X_1$ and produces the intermediate $O_i$, while the second complex $T_i$ binds the intermediate $O_i$ and produces the two species $X_2$ and $X_3$. The additional intermediate step is needed to ensure that the reactant species $X_1$ does not contain any overlapping domains with the product species $X_2$ and $X_3$. Note that the sequence of toehold domain $1_{q_i}$ can also be adjusted to be only partially complementary to domain 1, in order to tune the reaction rate $q_i$. (Color figure online)

Pioneering theoretical work (Soloveichik et al. 2010) showed how DNA could be used to implement a broad range of computation, including any computation that can be expressed as a chemical reaction network. The mechanism proposed was that of toehold-mediated DNA strand displacement (Zhang and Seelig 2011), whose systematic use was pioneered by Yurke et al. (2000). During this process, an invading single strand of DNA displaces an incumbent strand hybridized to a template (Fig. 4a). The process is mediated by a short exposed single-stranded region of DNA, referred to as a *toehold*. Various types of computation have been implemented experimentally using this approach, including elementary Boolean logic (Seelig et al. 2006), square root computation (Qian and Winfree 2011), neural network computation (Qian et al. 2011), distributed consensus capable of switching (Chen et al. 2013a), and oscillations (Srinivas et al. 2017).

**Fig. 5:**
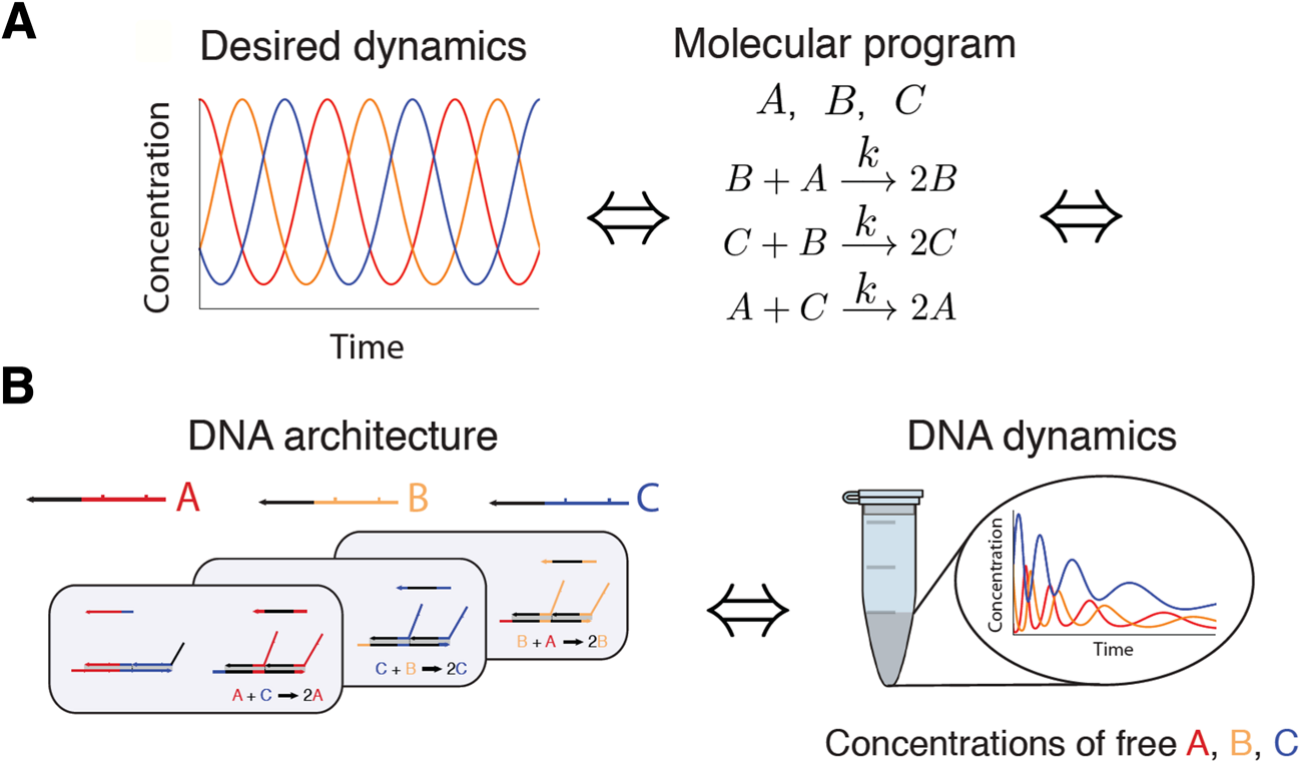
Implementation of an oscillator using a 4-domain DNA strand displacement scheme, reproduced from Srinivas et al. (2017). **a** The desired oscillatory dynamics are implemented by a molecular program, which is specified as a chemical reaction network. The network consists of three species (A, B, C) and three autocatalytic reactions, in which B converts A to itself, C converts B to itself, and A converts C to itself. This corresponds to the so-called rock-paper-scissors oscillator (Lachmann and Sella 1995). **b** The chemical reaction network is then translated to a 4-domain DNA architecture, similar to the one described in Fig. 4. Each molecular species is implemented as single DNA strand, and each reaction is implemented as a pair of DNA complexes together with an additional fuel strand. For example, the reaction $A + C\rightarrow 2A$ is implemented as a complex that consumes the A and C strands to produce an intermediate strand, and a complex that consumes the intermediate to produce two A strands, with the help of a fuel strand. The complexes and fuel strands are assumed to be present in excess and are consumed over time in a closed system, resulting in progressively slower oscillations

The general approach proposed by Soloveichik et al. (2010) for implementing an arbitrary chemical reaction network in DNA is based on a 4-domain scheme (Fig. 4). This approach was subsequently used by Srinivas et al. (2017) to implement an oscillator consisting solely of DNA (Fig. 5).

**Fig. 6:**
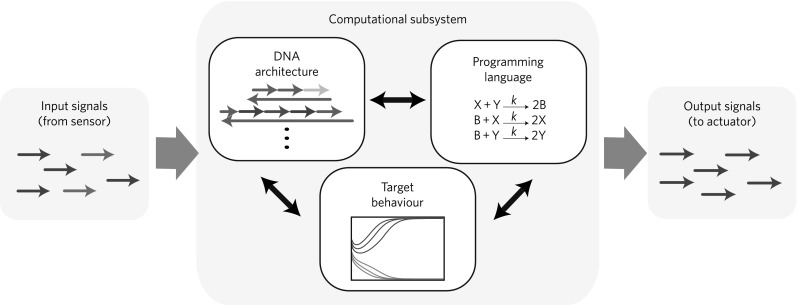
Implementation of a switch using a 2-domain DNA strand displacement encoding, reproduced from Chen et al. (2013a). The system takes as input two populations of signals, encoded as DNA strands, and uses a distributed consensus network to determine which population is in the majority. The output of the system is a homogeneous population of strands, in which all of the minority strands have been converted to the majority. As with the oscillator described in Fig. 5, the behaviour of the systems is specified as a chemical reaction network consisting of three reactions, in which X and Y cancel each other out to produce two intermediates B, species X converts B to itself, and species Y converts B to itself. This is equivalent to the Approximate Majority network (Angluin et al. 2008) described in Fig. 2

One of the potential drawbacks of the 4-domain scheme is that it requires synthetic DNA strands to be annealed. These synthetic strands can contain synthesis errors, which increase with strand length. A 2-domain DNA Strand Displacement scheme was proposed (Cardelli 2013), which enabled gates to be manufactured using plasmid DNA grown in cell culture. Since the DNA replication machinery of cells is substantially more accurate than existing DNA synthesis technology, particularly for long sequences, a large number of copies of the same double-stranded DNA sequence can be clonally replicated in cell culture. The culture is sequenced to check that no errors have been introduced and, since the population is clonal, if the sample sequence is correct then all copies of the sequence are also highly likely to be correct. The 2-domain scheme was used to implement the computational core of a switching network (Chen et al. 2013a) (Fig. 6).

Overall, one of the main advantages of using DNA strand displacement for the design and implementation of molecular-scale computation is its high degree of programmability, since all interactions are precisely encoded by the choice of DNA sequence. Moreover, system dynamics can be accurately predicted from computational models of their components (Chen et al. 2013a; Srinivas et al. 2017). Another important advantage is that the entire computation can be implemented solely in terms of DNA, without requiring additional enzymes. This simplifies system production, and also allows systems to be used in a broad range of biological contexts, with limited disruption. One of the main challenges is the need to replenish DNA strands and complexes in cases where dynamic behaviour needs to sustained for extended periods. To address this, complexes and fuel strands could be replenished periodically, or a system of buffered gates (Lakin et al. 2012) could be used. Another challenge is that unintended interactions between strands can lead to a decrease in system performance, for instance due to blunt-end strand displacement interactions that occur in the absence of a toehold, also known as *leaks*. One strategy for mitigating these leaks involves the use of toehold clamps (Qian and Winfree 2011), which can be used effectively in a systematic way (Wang et al. 2017). Another approach for reducing unwanted interference between DNA molecules more generally is to localise the molecules to DNA origami (Dalchau et al. 2015; Chatterjee et al. 2017), such that strands which are meant to interact are placed close to each other. This increases the local concentration of interacting strands, allowing fast computation, while reducing interference. See Yordanov et al. (2014) for a more in-depth discussion on the advantages and challenges of using DNA strand displacement in the context of implementing feedback controller systems, and a comparison with alternative nucleic acid implementation strategies.

### The polymerase-exonuclease-nickase dynamic network assembly (PEN-DNA) toolbox

**Fig. 7:**
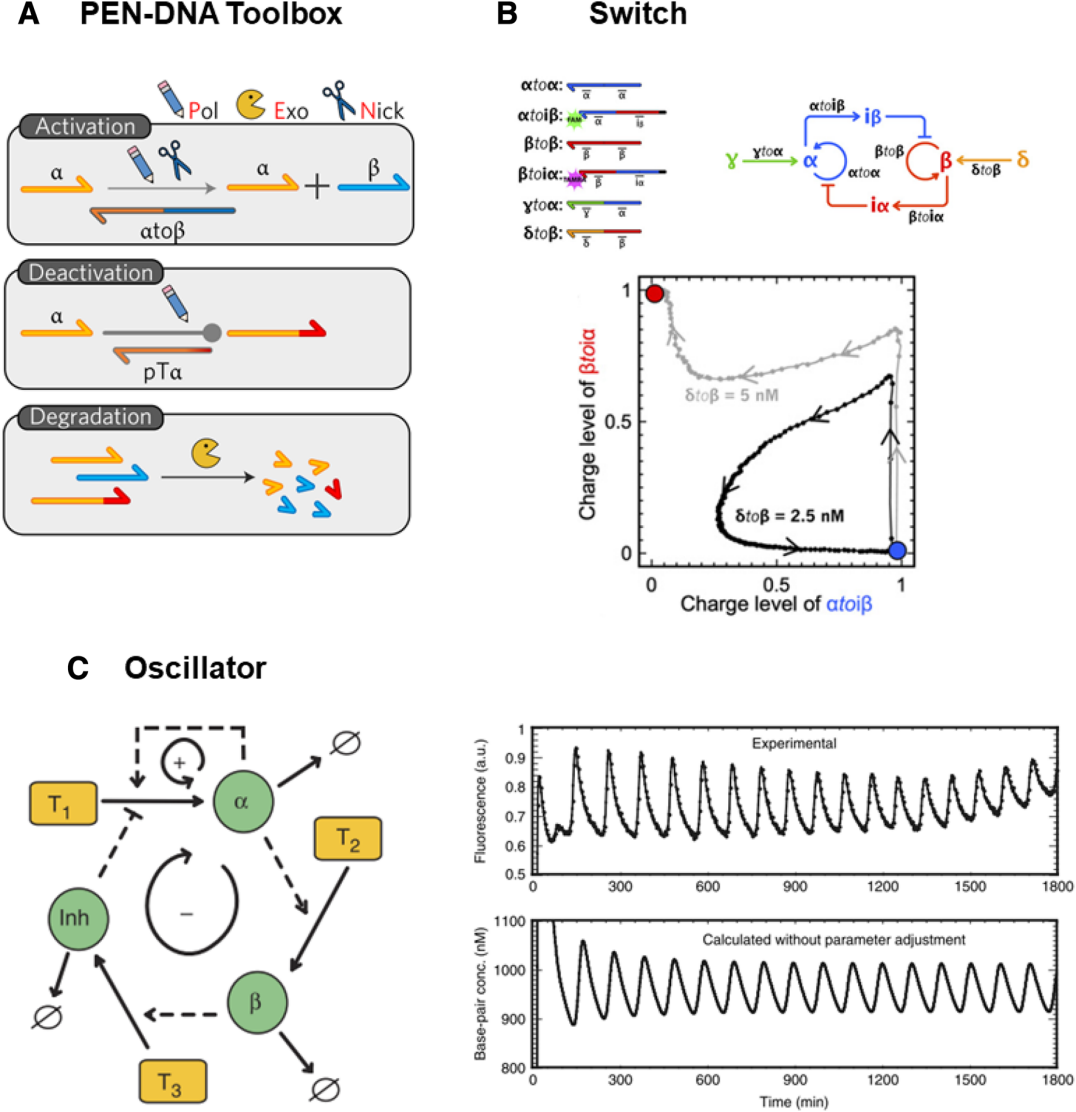
The PEN-DNA toolbox. **a** Summary of the PEN-DNA toolbox modules (reproduced from Meijer et al. 2017), with Polymerase (Pol), Exonuclease (Exo) and Nickase (Nick) enzymes. **b** The switchable memory circuit from Padirac et al. (2012). In the network diagram, blue symbols represent components associated with the high $\upalpha$-low $\upbeta$ state, red symbols represent the low $\upalpha$-high $\upbeta$ state, and green/orange symbols are the external inputs. Measurements correspond to treating with 2.5 nM or 5 nM of $\updelta to \upbeta$, as indicated. All graphics are reproduced from Padirac et al. (2012). **c** The negative feedback loop oscillator from Montagne et al. (2011). (Color figure online)

An alternative to DNA strand displacement for performing molecular computation uses enzymes to manipulate DNA signals. The PEN-DNA (Polymerase—Exonuclease—Nickase Dynamic Network Assembly) toolbox is a set of modules that can be composed to implement molecular programs (Fig. 7a) and, as we shall describe in this section, has successfully been used to implement switches and oscillators.

An *activation* module enables a short single-stranded DNA (ssDNA) signal $\alpha$ to stimulate production of another ssDNA signal $\upbeta$. This is achieved by a longer template strand $\upalpha\, to\,\upbeta$ composed of two consecutive domains, one complementary to $\upalpha$ and the other complementary to $\upbeta$. When $\upalpha$ binds to the 3’ side of the $\upalpha to \upbeta$ template, Polymerase is recruited, and elongates the input strand over the $\upbeta$ domain, producing a full duplex. The sequences of $\upalpha$ and $\upbeta$ are chosen to enable a Nickase enzyme to bind and convert the duplex into a nicked double-stranded molecule. This separates the upper domains, such that their affinity for the template is reduced and they can more easily unbind, leading to the release of the pre-existing $\upalpha$ and de novo synthesized $\upbeta$ ssDNA signals. As such, $\upalpha$ catalyses the production of $\upbeta$, analogous to the reaction $\upalpha \rightarrow \upalpha +\upbeta$. A *deactivation* module implements the reaction $\upalpha \rightarrow \upalpha ^\prime$, where $\upalpha ^\prime$ is notionally inactive. This is achieved by a pseudo-template pT$\upalpha$ that extends $\upalpha$ with a short oligonucleotide tail, preventing $\upalpha$ from participating in activation reactions. Finally, a 5’-3’ Exonuclease destroys all ssDNA signals, representing a non-specific *degradation* module.

#### Switches as memory devices

A bistable switch was one of the first circuits constructed using the PEN-DNA toolbox (Padirac et al. 2012) (Fig. 7b). The circuit design combined self-activation with mutual inhibition, resembling the MI network described above. The inner mutual inhibition module was achieved using four template strands: two templates implemented self-activation for $\upalpha$ ($\upalpha$ *to* $\upalpha$) and $\upbeta$ ($\upbeta$ *to* $\upbeta$), while a further two templates implemented inhibition with the production of inactive signals i$\upalpha$ and i$\upbeta$, which block $\upalpha$ and $\upbeta$ templates respectively. An additional two templates were then used to mediate inputs $\upgamma$ and $\updelta$ that could switch the device between the $\upalpha$ and $\upbeta$ states. The authors demonstrated that a high concentration of external input could switch the device in approximately 200 minutes, but with lower concentrations leading only to a transient excursion and then return to the pre-existing stable state. While the demonstration of the robustness of the switch is impressive, such a long switching time could prohibit its usage in some applications/ Nevertheless, by this circuit being both bistable and switchable, it can be used for long term memory storage. A push-push memory device was also constructed, which enabled switching back and forth in response to the same input signal.

Switches are fundamental building blocks for many computational devices, but their utilisation requires sensing of inputs and actuation. Recently, it was demonstrated how the PEN-DNA memory switch of Padirac et al. (2012) can be connected to downstream enzymatic actuators, enabling the connection of DNA-based memory devices to triggered downstream signalling (Meijer et al. 2017). To achieve this, a translator module was developed that dynamically perceives the short single-stranded DNA molecules of the bistable switchable memory device described above, then produces longer DNA strands that can be used to control the activation of two enzymes, NanoLuc and TEM1 $\beta$-lactamase. Importantly, the translator was designed to minimize retroactivity back to the memory switch, which was demonstrated with a detailed experimental and theoretical characterization. Finally, the activation/inactivation of the enzymes relies on interactions with conjugated oligonucleotides, which are modulated by the output of the translator module. This appears to be the first time memory devices and actuators have been connected in a synthetic molecular circuit, and is an important step towards realising more general molecular computers.

#### Limit cycle oscillators as clocks

The first attempt to produce an oscillator using the PEN toolbox approach was based on recapitulating a network topology that is known to robustly produce oscillatory behaviours (Montagne et al. 2011). Subsequently, another network architecture based on a predator-prey interaction was developed (Fujii and Rondelez 2013). Using both strategies, the Rondelez group were able to sustain oscillations for more than 10 cycles, with only a small amplitude loss. By taking advantage of molecular diffusion, and visualizing the solution between two glass slides, a follow-up work illustrated how these DNA-based oscillators can also produce travelling wave phenomena (Padirac et al. 2013).

Compared to oscillators constructed from purely DNA systems, the PEN-DNA systems exhibit temporal dynamics that can be sustained for substantially longer time periods. A feature of PEN-DNA that is likely to contribute to this is the ability to synthesize new copies of the signal strands. This is not possible with a purely strand displacement system, which produce *new* signal strands by releasing them from previously constructed gate species, supplied as fuel. Accordingly, the gate species are consumed over time and oscillator amplitudes drop. The PEN-DNA systems also exhibit changes in dynamics over time as the supply of nucleotides and other reagents is depleted, though this occurs on a longer time scale relative to the system dynamics.

## Future of biological computing

### Molecular programming in cells

Despite the limitations discussed above, there are considerable advantages to using DNA circuits to implement computation in cells. DNA offers a natural interface to the cellular machinery and is inherently biocompatible. After several years of exploring the computational potential of nucleic acid circuits in vitro, there are now efforts to deliver DNA circuits into live cells. In Groves et al. (2015), a variety of methods were compared for delivering nucleic acid circuits to mammalian (CHO and HeLa) cells, and it was shown that multi-input computation in live cells could be detected using flow cytometry. In the same study, chemical modifications to DNA and RNA strands were shown to improve binding kinetics, most likely a result of reduced nuclease activity against the modified strands. This has stimulated more detailed characterization studies of nuclease activity against nucleic acid circuits (Fern and Schulman 2017). Another fruitful strategy for delivering DNA circuits to live cells has been the use of DNA origami, which provides both a localizing and protective effect on circuit components, leading to faster circuit operation (Dalchau et al. 2015; Chatterjee et al. 2017), but also successful operation in a live animal (Amir et al. 2014).

Introducing molecular circuits based on the PEN-DNA toolbox into cells is made challenging by the need to express the PEN enzymes in the target cells. Enzymes impose several design constraints on the selection of DNA sequences. Nicking enzymes have specific recognition sites, which imposes a limit on the diversity of signal strands that can be used in a PEN-DNA circuit. In contrast, polymerase and exonuclease are non-specific, meaning that the activation and degradation reaction rates are difficult to control. While differential activation rates could be achieved by controlling activation template concentrations, dynamic behaviours that require differential degradation would be harder to engineer.

Nucleic acid circuits have the added benefit of requiring a reduced regulatory approval process compared with genetically modified organisms, for applications such as disease diagnosis and treatment. There have already been several attempts to use nucleic acids circuits, combined with transcriptional machinery, for biosensing and diagnosis (Pardee et al. 2016). Non-transcriptional nucleic acid circuits are in principle easier to program than transcriptional networks based on promoter regulation by proteins. In part, this is due to transcriptional control requiring the pairing of a protein surface with a DNA binding motif, an interaction that is challenging to engineer synthetically. However, new approaches based on CRISPR/dCas9 could lead to a more targeted way of engineering networks with precise topologies. Finally, nucleic acid circuits are a convenient test framework for programmed genetic circuits. They force the engineer to consider energy/substrate economy and the physical limitations of molecular binding, which, for example, is analogous to ribosomal usage and transcriptional and translational efficiency of synthetic gene circuits. Much of what we have learned in the design, characterization and analysis of nucleic acid circuits can be applied to the engineering of computational circuits based on other biomolecular frameworks.

### From molecular networks to algorithms

As we have seen above, algorithms that mimic the behaviour of biological switches and clocks can be implemented using DNA circuits, and the same dynamics can also be achieved using synthetic gene regulatory networks. It remains to be seen how more complex gene regulatory networks could implement more advanced algorithms, enabled by advanced genome editing techniques (Cong et al. 2013). More complex engineered networks of switches and clocks could also be combined with electronic circuits (Cao et al. 2017) to serve as biosensors. These applications could have a major influence on disease detection and treatment. However, to reach this stage requires a better understanding and control of elementary computing units. Thus, algorithmic thinking might be leveraged to detect and ultimately treat complex disease states, by combining switches and clocks with the existing logic circuit toolbox.

Historically, the analysis of biological mechanisms and collective behaviour from an algorithmic perspective led to simplified models, which aided understanding of information processing in natural systems. This laid the groundwork for future breakthroughs across disciplines (Marblestone et al. 2016; Navlakha and Bar-Joseph 2011; Whitley and Sutton 2012; Yang 2014). Such advances will continue, driven by the desire for scalability and robustness as the complexity of solid state technology approaches that of biological systems.

As precision in designing chemical systems increases, we look towards chemical computational units with which we may construct complex behaviours systematically. We have seen how computational DNA circuits and related technologies can be used as flexible molecular mechanisms to engineer switches, oscillators, and other computational components in vitro, with efforts being made also in vivo. Moreover, such computational units can be coupled with molecular sensors, actuators, and scaffolding to provide complete nanoscale devices. Many such devices and components can be individually designed and engineered using the DNA, RNA, and enzyme tricks of the biochemical trade.

### From single-cells to computational communities

Building on the fascinating advances in our ability to program cell-autonomous behaviours, there are now several examples of establishing behaviours that rely on multicellularity. Switches within individual cells can be linked via inter-cellular communication (see Hennig et al. 2015 for a thorough review), including natural quorum sensing molecules (Camilli and Bassler 2006) and artificial DNA messengers (Ortiz and Endy 2012; Goñi-Moreno et al. 2013). As such, it is possible to achieve behaviours of distributed computing algorithms with cells. Based on temporal logic, it was shown how cell populations could be used for timing and recording chemical events (Hsiao et al. 2016).

In additional to temporal control, intercellular communication also enables spatial control, and therefore programmed pattern formation. Already, this has enabled circuits that can detect spatial boundaries between an environmental signal (light) (Tabor et al. 2009) and establish stripe patterns in expanding colonies (Liu et al. 2011). More exploratory work with communicating bacterial populations has begun to shed light on how developmental patterns can be scale invariant (Cao et al. 2016), but also suggest a new platform for testing ecological theory, via synthetic ecosystems (Song et al. 2009). Furthermore, techniques such as live cell lithography can create regular structures of communicating microbes at resolutions of 5 $\mu$m (Mirsaidov et al. 2008). There is also work in controlling spatial distributions of DNA molecules directly (Dalchau et al. 2014; calise and Schulman 2014), which could be used to pre-pattern cellular systems.

Put together, there is now real promise for designing cell colonies that control their own temporal dynamics and spatial positioning. This could be highly relevant to biologics production in bioreactors, where spatial heterogeneity in resources and cell density could lead to inefficiencies. The advantages of automatic control can be readily seen in more traditional control engineering applications, and consequently, there is now rising interest of implementing control algorithms in biological circuits (Del Vecchio et al. 2016). Earlier strategies were based on transcriptional negative autoregulation (Becskei and Serrano 2000; Rosenfeld et al. 2002), but more recently, a more advanced *integral control* via negative feedback has been demonstrated in metabolic circuits (Briat and Khammash 2018), but also using optogenetics via in silico controllers (Milias-Argeitis et al. 2016; Lugagne et al. 2017). Automatic control will bring robust operation and self-adaptation to biological circuits, which will help to make biotechnology more efficient and ultimately more competitive in the marketplace (Del Vecchio et al. 2016).

Despite the promise of synthetic biology, significant challenges remain for regulating biological behaviours with the insertion of designed networks of transcriptional components. One challenge relates to timescale: transcriptional control is slow, though is the dominant mode of regulation in synthetic biology applications to date. Control algorithms are sometimes inefficient in the face of delays, and so their success might depend on establishing faster modes of biological regulation. Here, molecular programming might provide a solution, as DNA-based circuits can be localized, speeding up their operation (Chatterjee et al. 2017). Another challenge of using transcriptional regulation is that each additional component introduces a burden on the host cell, which can lead to poor growth of the colony, thus altering the performance of existing components and/or reduce yields (Scott et al. 2010; Borkowski et al. 2016; Wu et al. 2016). For example, inserting a component that leads to high levels of protein translation will impose a burden on the ribosome pool (Scott et al. 2010; Shachrai et al. 2010), high levels of transcription will impose a burden on RNA polymerase (Gyorgy et al. 2015), and high levels of protein degradation will impose a burden on proteases (Cookson et al. 2011). While some of these can be mitigated with quantification of the burden and careful design (Nielsen et al. 2016), establishing cellular control with components that do not consume/occupy shared cellular resources could be a major advantage. However, it remains unclear as to whether DNA circuits can operate reliably enough inside cells to rival existing approaches to cellular control.

The extent to which spatiotemporal control can improve biotechnology applications is relatively unexplored. Therefore, more theoretical work is needed to establish the performance that can be achieved by algorithms based on chemistry and cellular communication, and therefore to understand the capability of synthetic multicellular platforms.

### Learning from biological computing

As we increasingly rely on computing to process large datasets for everyday tasks at home and at work, power consumption will become a future limiting factor (Council 2011; Kamil et al. 2008). In fact the effects of thermodynamics—that is removing heat efficiently from semiconductor devices—has already driven the shift to multicore chips and parallel computing, which can improve on the performance scaling of single processors, but will fundamentally change how we develop programs (Council 2011). Biological systems perform computations at much lower levels of power consumption: estimates report 4 orders of magnitude for molecular machines (Nicolau et al. 2016) and up to 12 orders of magnitude for DNA computing (Adleman 1994). In addition, these computations are carried out in a robust manner, embedded within fluctuating environments, and often utilising components that are unreliable and noisy (Sarpeshkar 2010). This incredible performance is achieved through multi-scale hybrid analog and digital information processing. Biological computers have the additional advantage that they can interface directly with living systems and therefore open up new applications in biosensing with industrial and clinical relevance. For example, signal processing in the ear has already inspired electronics for low power cochlear implants (Mandal et al. 2009) and pattern recognition by neurons led to a novel analog-to-digital converter (Yang and Sarpeshkar 2006). These properties, combined with the high information storage density of DNA (Church et al. 2012; Goldman et al. 2013; Erlich and Zielinski 2017), provide exciting future directions for further research.

In this review we have highlighted how complex biomolecular networks make use of switches and oscillators to perform computation. The continued understanding of how this information processing is achieved at such high levels of robustness and low power requirements will require the concerted efforts of systems and synthetic biology in addition to leveraging tools from engineering and computer science. While it is unlikely that biological systems will ever replace silicon as our dominant computing platform, learning how they compute could have a significant impact on future computing architectures.
